# *Weizmannia coagulans* XY2 Mitigates Copper Neurotoxicity via Gut–Brain Axis Modulation of Tryptophan Metabolism and Oxidative‐Inflammatory Crosstalk

**DOI:** 10.1002/imo2.70066

**Published:** 2025-11-18

**Authors:** Yufang Gao, Xiaodong Zheng, Fujie Yan

**Affiliations:** ^1^ Department of Food Science and Nutrition, College of Biosystems Engineering and Food Science Zhejiang University Hangzhou China

## Abstract

Copper interferes with tryptophan metabolism and 5‐HT levels by modulating intestinal flora. Intestinal barrier breakdown and inflammatory response trigger nerve damage under copper exposure. *W. coagulans* XY2 alleviates copper‐induced neurotoxicity by targeting a multi‐dimensional “tryptophan metabolism‐antioxidant defense‐gut‐brain axis” network.

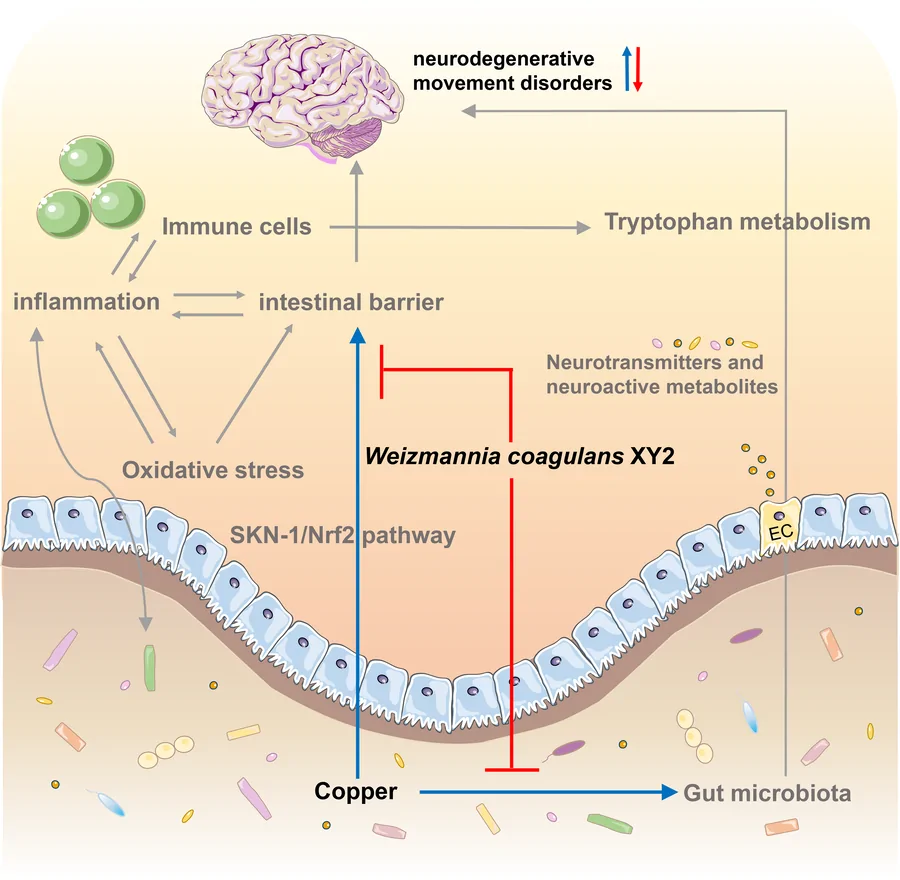

To the editor,

Copper pollution represents a significant and escalating global environmental concern driven by rapid industrialization and urbanization. While copper is an essential micronutrient for biological processes, its excessive accumulation poses serious health risks, particularly neurotoxicity and the acceleration of neurodegenerative diseases via trophic transfer [1, 2]. However, the precise mechanisms underlying copper‐induced neurotoxicity remain incompletely understood, and effective countermeasures are currently limited. This knowledge gap underscores the critical need to identify new therapeutic targets and develop safe strategies for mitigating copper toxicity.

This study elucidates a previously unrecognized gut–brain axis regulatory pathway mediated by copper‐triggered reprogramming of tryptophan (Tph) metabolism. Our principal findings are threefold: First, cerebral oxidative damage induced by copper exposure occurs not through direct brain copper accumulation, but rather originates from gut‐derived reactive oxygen species (ROS). These gut ROS activate the kynurenine pathway, diverting Tph metabolism towards the production of the neurotoxic metabolite quinolinic acid. Second, metabolomic profiling identified systemic disruption of Tph metabolism as a hallmark of copper neurotoxicity, characterized by depletion of serotonin and concomitant elevation of pro‐inflammatory cytokines. Third, 16S rRNA sequencing exposed copper‐mediated gut dysbiosis, particularly aberrant alterations in *Bifidobacterium* and *Akkermansia*, which are pivotal for Tph homeostasis. We further investigated the therapeutic potential of *Weizmannia coagulans* XY2, a probiotic previously isolated from coffee grounds in our laboratory [3]. Initially classified as *Bacillus coagulans* based on 16S rRNA gene sequencing and BLAST analysis, this strain has since been reclassified as *W. coagulans* following taxonomic revisions [3]. *W. coagulans* is recognized as an edible probiotic, characterized by its ability to produce lactic acid and form acid‐tolerant spores that facilitate intestinal colonization [4, 5, 6]. Therapeutically, *W. coagulans* XY2 exerts multidimensional neuroprotection by restoring gut microbiota‐mediated Tph metabolism, activating the SKN‐1/Nrf2 antioxidant pathway, and maintaining intestinal barrier integrity, thereby disrupting the detrimental “oxidative stress‐inflammation‐neurotoxicity” cascade initiated by copper. Our findings link environmental copper exposure to neurotoxicity via gut dysbiosis and Tph metabolic reprogramming. This study identifies the gut–brain metabolic axis as a pivotal target. Furthermore, we demonstrate the efficacy of *W. coagulans* XY2 as a probiotic‐based therapeutic strategy for preventing copper‐induced neurotoxicity, offering a promising and safe intervention approach.

## 1 MODULATION OF COPPER‐INDUCED CEREBRAL METABOLIC DISORDERS BY *WEIZMANNIA COAGULANS* XY2 IN MICE

Nontargeted metabolomics of murine brain tissue revealed profound copper‐induced metabolic dysregulation, with 147 differentially abundant metabolites (80 upregulated, 67 downregulated; *p* < 0.05, VIP > 1) and significant perturbations in neural/oxidative pathways, including dopaminergic/GABAergic synapses, TCA cycle, and Parkinson's disease (Figure 1A, Figure S1). These findings collectively demonstrate profound copper‐induced disruption of cerebral metabolism.

**Figure 1:**
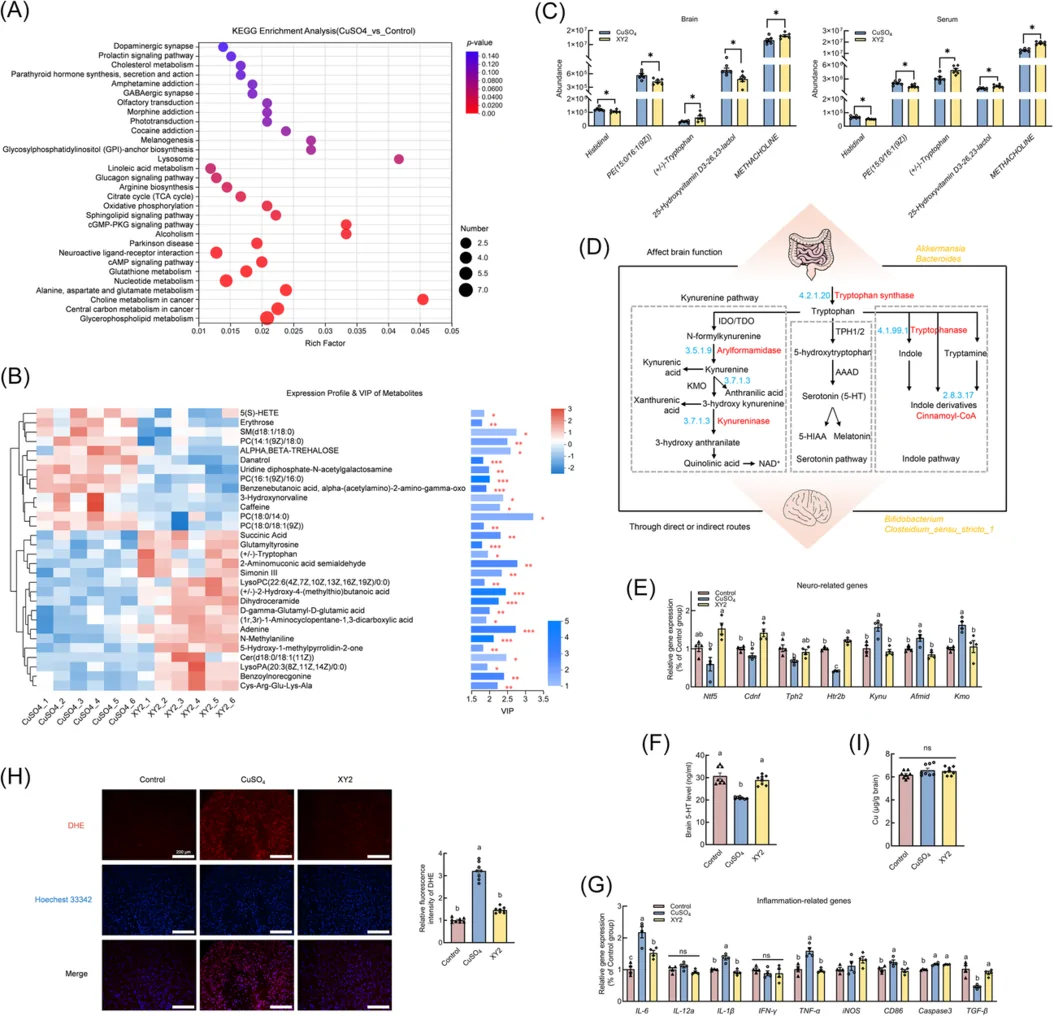
Tph metabolism was a key pathway for *W. coagulans* XY2 to inhibit copper toxicity. (A) Bubble chart of top 30 KEGG pathway enrichment analysis involving differential metabolites between CuSO_4_ group and Control group. Larger bubbles indicate more metabolites in the pathway, while a redder color indicates a smaller *p*‐value. Six mice were examined per treatment. (B) Clustering heatmap and Variable importance in projection (VIP) scores of the top 30 differential metabolites between XY2 and CuSO_4_ groups. Asterisk represented a significant difference between CuSO_4_ group and XY2 group (*p < 0.05, ** *p* < 0.01, *** *p* < 0.001, unpaired two‐tailed Student's *t*‐tests, *n* = 6). (C) Abundance in the brain and serum of overlapping differential metabolites of serum and brain metabolomes. Asterisk represented a significant difference between CuSO_4_ group and XY2 group (*p < 0.05, unpaired two‐tailed Student's *t*‐tests, *n* = 6). (D) Network diagram of Tph metabolism. Black font represents metabolites in the Tph pathway, blue font represents EC numbers of involved enzymes, red font represents functional domains of involved enzymes, and yellow font represents Tph‐associated differential gut flora. IDO = indoleamine‐2,3‐dioxygenase, TDO = Tph‐2,3‐dioxygenase, KMO = Kynurenine 3‐monooxygenase, AAAD = aromatic amino acid decarboxylase, TPH1/2 = tryptophan hydroxylase1/2, 5‐HIAA = 5‐hydroxyindoleacetic acid. (E) mRNA expression levels of neurologically relevant genes. Different letters represented significant differences among experimental groups, while the same letters represent no significant differences (*p* < 0.05, one‐way ANOVA followed by Tukey test, *n* = 4). (F) Brain 5‐HT level. Different letters represented significant differences among experimental groups, while the same letters represent no significant differences (*p* < 0.05, one‐way ANOVA followed by Tukey test, *n* = 8). (G) mRNA expression levels of inflammation‐related genes. Different letters represented significant differences among experimental groups, while the same letters and ns represent no significant differences (*p* < 0.05, one‐way ANOVA followed by Tukey test, *n* = 4). (H) Representative images of O_2_^−^ levels in brain tissue and the relative fluorescence intensity of O_2_^−^. Scale bars = 200 μm. Different letters represented significant differences among experimental groups, while the same letters represent no significant differences (*p* < 0.05, one‐way ANOVA followed by Tukey test, *n* = 8). (I) Copper levels in the brain. ns, not significant (*p* < 0.05, one‐way ANOVA followed by Tukey test, *n* = 8).

*W. coagulans* XY2 intervention primarily affected “lipids and lipid‐like molecules” (33.00%, Figure S2A). The most enriched KEGG pathways were lipid metabolism, cancer: general overview, amino acid metabolism, and nervous system (Figure S2B). Topological analysis identified the most significant metabolic pathways: Glycerophospholipid metabolism, Sphingolipid metabolism, Vitamin B6 metabolism, Histidine metabolism, and Caffeine metabolism (Figure S2C). Critically, *W. coagulans* XY2 intervention significantly reversed specific copper‐induced lipidomic imbalances. It markedly reduced the levels of PC(14:1(9Z)/18:0), PC(16:1(9Z)/16:0), PC(18:0/14:0), PC(18:0/18:1(9Z)), PC(18:3(9Z,12Z,15Z)/18:1(11Z)), PE(15:0/16:1(9Z)), Glycerylphosphorylcholine and Caffeine, while significantly increasing levels of LysoPC(18:0/0:0), LysoPC(22:6 (4Z,7Z,10Z,13Z,16Z,19Z)/0:0), l‐histidine, Dihydroceramide and d‐Erythrose‐4‐Phosphate (Figure 1B). This metabolic reprogramming restores cerebral lipid homeostasis and functionally correlates with attenuated oxidative stress and improved neurological outcomes.

## 2 TRYPTOPHAN METABOLISM IS A KEY PATHWAY FOR *W. COAGULANS* XY2 TO INHIBIT COPPER TOXICITY

Serum metabolomics analysis showed that the CuSO_4_ group exhibited 34 upregulated and 29 downregulated metabolites compared to the Control group, while *W. coagulans* XY2 intervention resulted in 41 upregulated and 51 downregulated metabolites relative to the CuSO_4_ group (Figure S3A). Five common differential metabolites were found in both brain and serum: Histidinal, PE(15:0/16:1(9Z)), (+/−)‐Tph, 25‐Hydroxyvitamin D3‐26,23‐lactol, and METHACHOLINE (Figure 1C). Disruptions in Tph metabolism may impact neurological diseases and are linked to changes in gut microbiota [7]. We further explored three key Tph metabolic pathways (Figure 1D).

Serotonin pathway: Enterochromaffin cells synthesize approximately 90% of the body's 5‐hydroxytryptamine (5‐HT) via TPH1, while neuronal 5‐HT synthesis in the central nervous system and enteric nervous system is mediated by neuron‐specific TPH2. 5‐HT is crucial for central neurotransmission and gastrointestinal functions. Copper exposure significantly downregulated *Tph2* expression and reduced cerebral 5‐HT levels, whereas *W. coagulans* XY2 intervention reversed these alterations (Figure 1E,F).

Kynurenine pathway: Copper exposure upregulated key genes (Kynureninase (*Kynu*), Arylformamidase (*Afmid*), and kynurenine 3‐monooxygenase (*Kmo*) encode the vital enzymes Kynureninase (EC 3.7.1.3), Arylformamidase (EC 3.5.1.9), and KMO, respectively), promoting the conversion of Tph to the neurotoxic quinolinic acid, while *W. coagulans* XY2 significantly reduced the expression of these genes and quinolinic acid production (Figure 1E). Pro‐inflammatory cytokines trigger neurotoxicity by activating indoleamine 2,3‐dioxygenase and KMO to accelerate Tph conversion into kynurenine and quinolinic acid [8]. In this study, copper intake significantly elevated pro‐inflammatory cytokine expression (Interleukin‐6 (*IL‐6*), Interleukin‐1 beta (*IL‐1β*), Tumor Necrosis Factor alpha (*TNF‐α*)), whereas *W. coagulans* XY2 markedly suppressed their expression, thereby disrupting the inflammation‐oxidative stress‐neural damage vicious cycle (Figure 1G).

Indole pathway: Gut microbiota metabolize unabsorbed Tph into indole and its derivatives to regulate central nervous system function. 16S rRNA sequencing revealed that *W. coagulans* XY2 reversed copper‐induced aberrant elevation in *Bifidobacterium* and *Akkermansia* abundance (Figure S3B). Previous studies confirm that *Bifidobacterium adolescentis* converted Tph into indole derivatives [9], while *Akkermansia muciniphila* and its extracellular vesicles modulated 5‐HT levels via the gut–brain axis [10]. Furthermore, *W. coagulans* XY2 significantly increased the abundance of *Clostridium_sensu_stricto_1*, which regulated enterochromaffin cell development and 5‐HT release [11].

PICRUSt2 analysis predicted that copper exposure may disrupt key enzymes in Tph metabolism, including EC 3.5.1.9, EC 3.7.1.3, Tph synthase (EC 4.2.1.20), Tryptophanase (EC 4.1.99.1), and Cinnamoyl‐CoA: phenyllactate CoA‐transferase (EC 2.8.3.17) (Figure S3C). These predictions will be further validated in future studies through metabolomics and metagenomics analyses.

The study further revealed that copper exposure significantly downregulated gene expression of Neurotrophin 5 (*Ntf5*) and Serotonin receptor 2B (*Htr2b*) in mouse brains, concurrently reducing the anti‐inflammatory factor Transforming Growth Factor beta (*TGF‐β*) (Figure 1E,G). *W. coagulans* XY2 intervention restored normal expression of these genes, indicating its capacity to ameliorate copper‐induced neural damage and inflammatory responses. Copper exposure also increased Superoxide Anion (O_2_^−^) levels and decreased antioxidant enzyme activities in the mouse brain, which were restored toward normal by *W. coagulans* XY2 (Figure 1H, Figure S4A). Notably, no significant differences in brain copper content were observed, suggesting indirect neurotoxicity via the gut–brain axis rather than direct brain effects (Figure 1I).

## 3 *W. COAGULANS* XY2 INHIBITED COPPER‐INDUCED NEUROTOXICITY THROUGH THE GUT–BRAIN AXIS

Copper exposure elevated colonic O_2_^−^ while decreasing glutathione reductase (GR) and catalase (CAT) activities, paradoxically increasing glutathione (GSH) and glutathione peroxidase (GSH‐Px) as compensatory responses (Figure S4B,C). *W. coagulans* XY2 intervention restored these oxidative parameters toward normal. Copper‐induced oxidative stress is closely associated with the Nuclear factor erythroid 2‐related factor 2 (Nrf2) pathway and the Forkhead box protein O1 (FoxO1) pathway [12]. Furthermore, a mutually reinforcing cascade exists between inflammation and oxidative stress. Crucially, *W. coagulans* XY2 reversed copper‐induced Nrf2 pathway hyperactivation, including *Nrf2*, Microsomal glutathione S‐transferase 1 (*Gst*), Glutathione S‐transferase Pi 1 (*Gstp1*), *Cu/Zn‐SOD*, *Mn‐SOD*, *GSH‐Px*, *GR* and suppressed pro‐inflammatory mediators (*IL‐6*, Interleukin‐12 subunit alpha (*IL‐12a*), *IL‐1β*, Interferon gamma (*IFN‐γ*), *TNF‐α*, Cluster of Differentiation 86 (*CD86*), *Caspase3*), while resolving the paradoxical elevation of *TGF‐β* (Figure 2A,B). Furthermore, *W. coagulans* XY2 rescued copper‐depleted Tph1 and 5‐HT by 270.33% and 448.29%, respectively (Figure 2C,D), and restored intestinal barrier integrity through upregulation of tight junction proteins (Claudin‐2/3 (*Cldn2/3*), Gap Junction Protein Alpha 1 (*Gja1*), and Mucin 2 (*Muc2*)) (Figure 2E, Figure S5). This integrated mitigation of oxidative stress, inflammation, and barrier dysfunction underpins the strain's neuroprotective efficacy.

**Figure 2:**
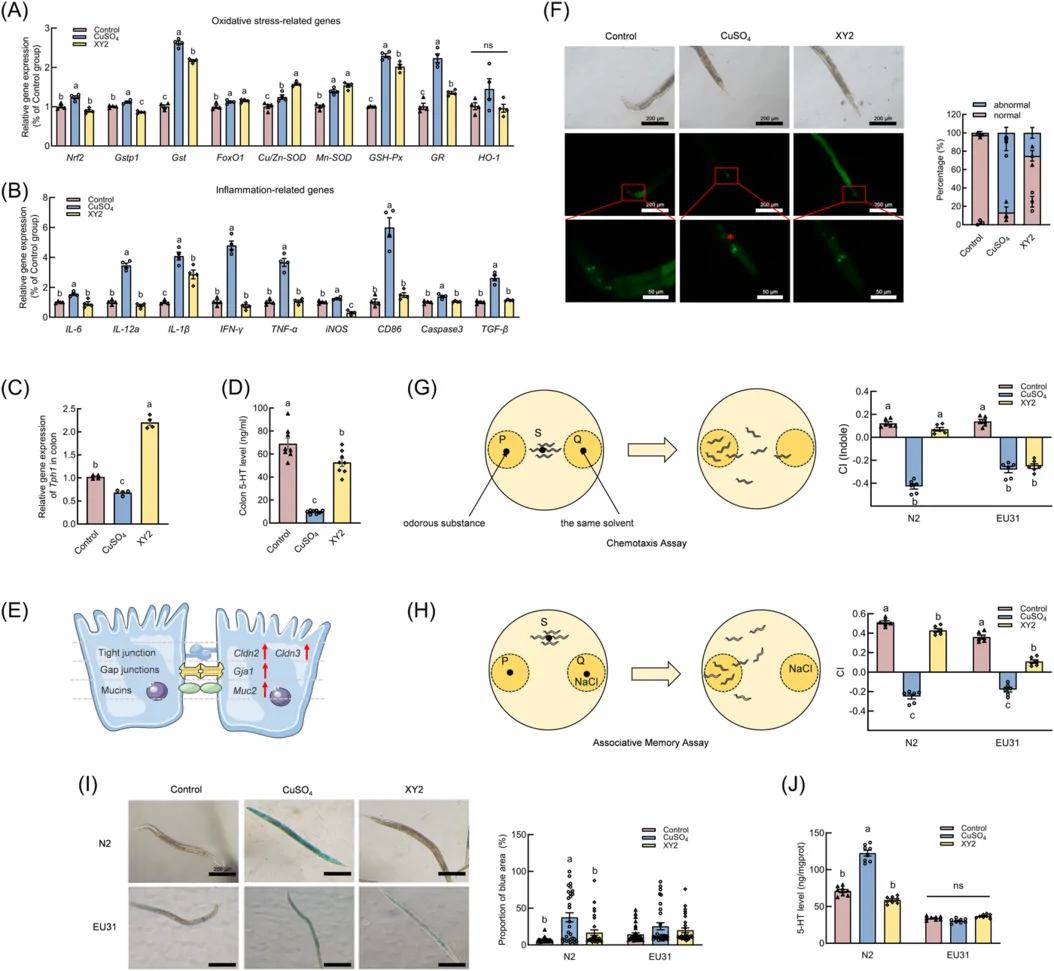
*W. coagulans* XY2 inhibited copper‐induced neurotoxicity through the gut–brain axis. (A) mRNA expression levels of oxidative‐related genes. Different letters represented significant differences among experimental groups, while the same letters represent no significant differences (*p* < 0.05, one‐way ANOVA followed by Tukey test, *n* = 4). (B) mRNA expression levels of inflammation‐related genes. Different letters represented significant differences among experimental groups, while the same letters represent no significant differences (*p* < 0.05, one‐way ANOVA followed by Tukey test, *n* = 4). (C) mRNA expression levels of *Tph1*. Different letters represented significant differences among experimental groups, while the same letters represent no significant differences (*p* < 0.05, one‐way ANOVA followed by Tukey test, *n* = 4). (D) Colon 5‐HT level. Different letters represented significant differences among experimental groups, while the same letters represent no significant differences (*p* < 0.05, one‐way ANOVA followed by Tukey test, *n* = 8). (E) Diagram of the genes involved in the intestinal barrier. The red arrow indicates that *W. coagulans* XY2 treatment significantly upregulated gene expression compared with the CuSO_4_ group (*p* < 0.05, one‐way ANOVA followed by Tukey test, *n* = 4). (F) Representative pictures of the serotonergic in GR1366 and the proportion of injured serotonergic neurons. Asterisks represent neuronal loss. Scale bars = 200 μm for low magnification and 50 μm for high magnification. Experiments were conducted in triplicate, and at least 30 nematodes were observed each time. (G) Schematic of chemotaxis assay and chemotaxis behavior to indole in N2 and EU31. Add 5ul of an odorous substance and solvent in locations P and Q equidistant from the center of the 9 cm NGM petri dish, then add the nematode at the center point S. After 1 h, nematode counts were taken in circles with a 3 cm diameter centered on points P and Q. Different letters represented significant differences among experimental groups, while the same letters represent no significant differences (*p* < 0.05, one‐way ANOVA followed by Tukey test). At least 100 nematodes were present in each group, and 6 replicates were conducted. (H) Schematic of associative memory assay and associative memory behavior in N2 and EU31. Two points, P and Q, were 4 cm apart. At Q, 1 cm^3^ NGM medium containing 100 mM NaCl was added at Q. The 9 cm NGM petri dish was refrigerated at 4°C overnight to create a NaCl gradient. The medium was taken out 15 min before positioning hungry nematodes at point S, 3 cm away from P and Q. After 1 h, nematode counts were taken in circles with a 3 cm diameter centered on points P and Q. Different letters represented significant differences among experimental groups, while the same letters represent no significant differences (*p* < 0.05, one‐way ANOVA followed by Tukey test). At least 100 nematodes were present in each group, and 6six replicates were conducted. (I) Representative pictures of intestinal permeability in N2 and EU31 nematodes and the proportion of blue area. Scale bars = 200 μm. Different letters represented significant differences among experimental groups, while the same letters represent no significant differences (*p* < 0.05, one‐way ANOVA followed by Tukey test, *n* = 30). (J) 5‐HT level in N2 and EU31. Different letters represented significant differences among experimental groups, while the same letters and ns represent no significant differences (*p* < 0.05, one‐way ANOVA followed by Tukey test). At least 100 nematodes were present in each group, and six replicates were conducted.

Gut microbiota significantly influences neurotoxicity via complex gut–brain axis pathways. Firstly, microbiota regulate microbial metabolites and neurotransmitters. For example, time‐restricted feeding enhanced *Bifidobacterium pseudolongum*'s production of propionic acid, improving cognition in Alzheimer's mice by activating free fatty acid receptor 3 [13]. Secondly, it modulates the intestinal barrier. *Holdemanella* upregulated tight junction proteins, but its neurological role is debated‐linked to both protection against depression/anxiety and associations with autism spectrum disorder and diabetes‐related cognitive impairment [14]. Thirdly, gut bacteria release extracellular vesicles carrying proteins and small RNAs. These vesicles can cross the blood‐brain barrier directly, delivering signals to brain cells that may either promote neurological diseases or suppress neurotoxicity [15]. These paradoxical findings highlight the communication complexity. Future research should prioritize exploring microbiota modulation as a therapeutic strategy to mitigate neurotoxicity and improve clinical outcomes in neurological disorders.

## 4 SKN‐1/NRF2 PATHWAY WAS CRITICAL FOR *W. COAGULANS* XY2 TO MITIGATE COPPER‐INDUCED NEUROTOXICITY

Transgenic nematode strain GR1366 has two pairs of neurons, NSM and ADF, with similar *tph‐1* expression levels, which are important for 5‐HT synthesis. As a dynamic neuron, ADF neurons are closely related to the learning association behavior of nematodes. 86.67% of nematodes in the CuSO_4_ group had significant damage and absence of ADF neurons, reduced to 25.00% with *W. coagulans* XY2 intake (Figure 2F).

*Caenorhabditis elegans* model validation demonstrated that copper exposure reduced head swing frequency by 34.53% and body bend frequency by 51.86% in wild‐type (N2) nematodes. However, *W. coagulans* XY2 restored these levels toward normal (Figure S6A,B). Normally, nematodes move closer to the presence of indole, but copper ingestion caused the exact opposite to occur (Figure 2G). Nematodes consistently exposed to high salt and food shortage simultaneously were able to form specific associative memories, and they will avoid exposure to NaCl if they encounter food shortages again. However, the worms in the CuSO_4_ group did not show a rejection of NaCl, suggesting that copper affected the learning and memory ability (Figure 2H). Nematodes in the XY2 group recovered their repulsive effect on NaCl and their chemotaxis towards indole, aligning with the previous GR1366 result. In summary, *W. coagulans* XY2 significantly inhibited copper‐induced neurodegenerative movement disorders.

Intestinal barrier integrity assessment revealed that copper exposure caused intestinal barrier damage in 48.00% of nematodes, with the brilliant blue dye‐positive area increasing from 6.61% to 41.21%. Following XY2 intervention, the proportion of damaged nematodes significantly decreased, and the dye‐positive area reduced to 16.72% (Figure 2I). Skinhead‐1 (SKN‐1) is the nematode homologue of the mammalian Nrf2 transcription factor. Validation using skn‐1(‐) mutant (EU31) demonstrated that *W. coagulans* XY2 failed to improve abnormal indole chemotaxis, intestinal barrier damage, and dysregulated 5‐HT levels in EU31, meanwhile its ameliorative effect on associative memory capacity was significantly attenuated, demonstrating SKN‐1/Nrf2 pathway‐dependent protection (Figure 2G–J).

The SKN‐1/Nrf2 pathway played a pivotal role in probiotic intervention. The potential crosstalk and interaction between this pathway and other signaling cascades within the gut microbiota‐brain axis warrant in‐depth investigation. Short‐chain fatty acids (SCFAs), as key metabolites derived from gut microbiota, modulated redox homeostasis via the Kelch‐like ECH‐associated protein 1 (Keap1)‐Nrf2 signaling axis, promoting Nrf2 nuclear translocation and transcriptional activation, thereby exerting synergistic antioxidant and anti‐inflammatory effects [16]. Studies demonstrate that SCFAs synergize with the Nrf2/Heme Oxygenase‐1 (HO‐1) pathway to protect the intestinal barrier in mice with acute ulcerative colitis [17]. As a critical component of the gut–brain axis, vagal nerve stimulation elevated cerebral Nrf2 levels and reduced pro‐inflammatory cytokines. The underlying mechanism involved the vagus nerve targeting the α7 nicotinic acetylcholine receptor, subsequently activating the Nrf2/HO‐1 pathway and ameliorating cognitive dysfunction [18]. Collectively, the crosstalk and interactions between the SKN‐1/Nrf2 pathway and other gut–brain axis pathways exhibit considerable complexity. This intricate interplay is likely crucial for maintaining gut–brain homeostasis and achieving effective probiotic modulation. Future research will focus on elucidating the relationships between these pathways to gain a more comprehensive understanding of the mechanisms underlying probiotic intervention.

This study establishes that *W. coagulans* XY2 confers neuroprotection against copper toxicity through a gut–brain metabolic axis. It regulates Tph metabolism, enhances antioxidant defense, and maintains intestinal barrier integrity. *W. coagulans* XY2 boosts serotonin production, inhibits the neurotoxic kynurenine pathway, and promotes neuroprotective indole production to improve neural function. It also activates the SKN‐1/Nrf2 antioxidant pathway and preserves intestinal barrier function to prevent oxidative‐inflammatory damage. These findings provide experimental evidence for probiotic‐mediated heavy metal detoxification via gut microbes‐derived neurotransmitter‐brain crosstalk, extending beyond established models of copper‐induced mitochondrial dysfunction. By defining the gut–brain axis as a therapeutic target and pioneering the repurposing of probiotics for environmental neurotoxicity management, this study delivers a safe, multi‐targeted strategy against copper‐driven neurodegeneration with significant translational potential.

## AUTHOR CONTRIBUTIONS

**Yufang Gao**: Investigation; formal analysis; methodology; software; visualization; writing–original draft. **Xiaodong Zheng**: Resources; supervision. **Fujie Yan**: Conceptualization; supervision; project administration; writing–review and editing. All authors have read the final manuscript and approved it for publication.

## CONFLICT OF INTEREST STATEMENT

The authors declare no conflicts of interest.

## ETHICS STATEMENT

The ethics application was approved by the Laboratory Animal Welfare and Ethics Committee of Zhejiang University (ZJU20220045).

## Supporting information


**Figure S1.** Copper caused metabolic disorders in the mouse brain.
**Figure S2.** *W*. *coagulans* XY2 modulated copper‐induced metabolic disorders in the mouse brain.
**Figure S3.** Tph metabolism‐related parameters.
**Figure S4.** *W*. *coagulans* XY2 attenuated copper‐induced oxidative stress in the brain and colon of mice.
**Figure S5.** mRNA expression levels of intestinal barrier‐related genes.
**Figure S6.** *W*. *coagulans* XY2 inhibited copper‐induced neurodegenerative movement disorders in N2 nematodes.

**Table S1.** The primers used in the mouse experiment.
**Table S2.** Abbreviation.

## Data Availability

Raw sequencing data on C57BL/6J mice have been saved in the Sequence Read Archive (SRA) database https://www.ncbi.nlm.nih.gov/bioproject/PRJNA896555. The data used is saved in GitHub https://github.com/GYF0429/Copper-Neurotoxicity/tree/master. Supplementary materials (methods, figures, tables, graphical abstract, slides, videos, Chinese translated version, and update materials) may be found in the online DOI or iMetaScience http://www.imeta.science/imetaomics/.
