# Supplementary material for: Weizmannia coagulans XY2 Mitigates Copper Neurotoxicity via Gut–Brain Axis Modulation of Tryptophan Metabolism and Oxidative‐Inflammatory Crosstalk
Source: IMetaOmics. 2025 Nov 18;3(1):e70066. doi: 10.1002/imo2.70066 (PMC13674420; doi:10.1002/imo2.70066)
Supplement: Supplementary file 1 [file IMO2-3-e70066-s002.docx]

**Supporting information to *Weizmannia coagulans* XY2 Mitigates Copper Neurotoxicity via Gut-Brain Axis Modulation of Tryptophan Metabolism and Oxidative-Inflammatory Crosstalk**

**Running title**: Gut-brain axis disorder promotes copper-induced neurotoxicity

Yufang Gao, Xiaodong Zheng, Fujie Yan*

Department of Food Science and Nutrition, College of Biosystems Engineering and Food Science, Zhejiang University, Hangzhou 310058, China.

***Correspondence**: [fjyan@zju.edu.cn](mailto:fjyan@zju.edu.cn) (Fujie Yan)

Figure S1 Copper caused metabolic disorders in the mouse brain.

Figure S2 *W. coagulans* XY2 modulated copper-induced metabolic disorders in mouse brain.

Figure S3 Tph metabolism-related parameters.

Figure S4 *W. coagulans* XY2 attenuated copper-induced oxidative stress in the brain and colon of mice.

Figure S5 mRNA expression levels of intestinal barrier-related genes.

Figure S6 *W. coagulans* XY2 inhibited copper-induced neurodegenerative movement disorders in N2 nematodes.

Table S1 The primers used in the mouse experiment (show in supplementary table excel file).

Table S2 Abbreviation (show in supplementary table excel file).

**MATERIALS AND METHODS**

**Strains and cultures**

*W. coagulans* XY2 was derived from fermented coffee grounds [1]. We acquired *Escherichia coli* OP50 from the China General Microbiological Culture Collection Center. For subsequent experiments, they were subjected to three activation steps, and the specific procedures are as follows:

*W. coagulans* XY2: First, it was inoculated on De Man, Rogosa, and Sharpe medium (MRS) agar plates and incubated at 50 °C. After 24 hours, a single colony was picked and inoculated into 5 mL MRS liquid medium for 18 h of cultivation, then transferred to 100 mL MRS liquid medium at an inoculum size of 2% for expanded cultivation for 18 h.

*E. coli* OP50: It was inoculated on Luria-Bertani (LB) agar plates and incubated at 37 °C. After 24 hours, a single colony was picked and inoculated into 5 mL LB liquid medium for 18 h of cultivation, then transferred to 100 mL LB liquid medium at an inoculum size of 2% for expanded cultivation for 18 h.

After the above cultivation, the bacterial pellets were collected by centrifugation (4 °C, 7000 g for 10 minutes) and washed twice with sterile PBS to remove residual medium components.

**Mice and experimental design**

We purchased 24 8-week-old male C57BL/6J mice from SLAC Laboratory Animal Co. Ltd (Shanghai, China). After one week of adaptive feeding (temperature maintained at 21-25 °C, relative humidity 48-55%, 12-hour circadian rhythm), the mice were randomly assigned to 3 groups of 8 mice for a 6-week experiment with the following design: Control group: drinking distilled water and gavage pure water. CuSO_4_ group: distilled water was consumed for the first two weeks, purified water was intragastric, and water added with copper sulfate (0.5 g/L) was consumed from the third week [2]. XY2 group: distilled water was consumed for the first two weeks, *W. coagulans* XY2 (10^10^ CFU/kg body weight) was intragastric, and water added with copper sulfate (0.5 g/L) was consumed from the third week. Both drinking water replacement and gavage (0.4 mL) were performed once every two days during the experiment, and all mice were fed standard normal diets (The copper content was 16.18 mg/kg) obtained from Zhejiang laboratory animal center (Hangzhou, China). Water and food were given to the mice ad libitum.

At the end of the experiment, the mice were fasted for 12 hours and euthanized by CO_2_ asphyxiation, and then blood, cecum contents, brain tissue and liver tissue were collected. This work has received approval for research ethics from Laboratory Animal Welfare and Ethics Committee of Zhejiang University (ZJU20220045) and a proof of approval is available upon request. During the experiment, we strictly followed all relevant guidelines and regulations.

**Fluorescent staining**

According to established protocols, hippocampus and colon tissues were cut into 6 μm sections using a cryosectioner at -20 °C [3]. The sections were washed with PBS twice and incubated with 1 mL fluorescent probe at room temperature for 10 min. Superoxide Anion (O_2_^-^) probe configuration: 2.5 μg/mL Dihydroethidium (DHE), 1 μg/mL Hoechst 33342. Rinse the slide with PBS, add 5 μL 5% buffer glycerin on top of the slide, and cover the cover glass. Following the staining process, the fluorescence microscope captured red and blue fluorescence through the FITC and UV channels, respectively, all under identical conditions. 8 mice were examined per treatment.

**Metabolomics**

Brain and serum metabolomics were performed using an untargeted metabolomics approach [4]. Serum sample processing: 6 mouse blood was centrifuged (3,000 × g, 15 min, 4 °C), and the supernatant was collected and stored at -80°C. Prior to analysis, serum samples (100 µL) were mixed with acetonitrile/methanol (1:1, v/v), vortexed for 30 s, and subjected to low-temperature ultrasonication (5 °C, 40 kHz) for 30 min. Samples were placed at -20°C for 30 min, followed by centrifugation (13,000 × g, 15 min, 4 °C). The supernatant was transferred and evaporated under a nitrogen stream. The residue was reconstituted in 100 µL acetonitrile/water (1:1, v/v), after which the extraction and centrifugation steps were repeated. The final supernatant was transferred to autosampler vials with inserts for instrumental analysis. Brain tissue processing: 6 brain tissue samples (100 mg) were placed in 2 mL microtubes with a 6-mm grinding bead and 800 μL methanol/water (4:1, v/v) for metabolite extraction. Homogenization was performed in a cryogenic tissue grinder (-10°C, 50 Hz) for 6 min, followed by low-temperature ultrasonication (5 °C, 40 kHz) for 30 min. Samples were placed at -20°C for 30 min and centrifuged (13,000 × g, 15 min, 4 °C). The supernatant was directly transferred to autosampler vials with inserts for analysis. Quantitative analysis of extracts using Thermo Fisher Scientific's UHPLC-Q Exactive HF-X system and HSS T3 column (100 mm x 2.1 mm, 1.8 μm). The mobile phase consisted of 0.1% formic acid in acetonitrile/ H_2_O (5:95, v/v; phase A) and 0.1% formic acid in [isopropanol](javascript:;)/acetonitrile/H_2_O (47.5:47.5:5, v/v/v; phase B). The flow rate was 0.40 mL/min, and the column temperature was 40 ℃. The injection volume was 5 μL. The UHPLC system was coupled to a UHPLC-Q Exactive HF-X system Mass Spectrometer equipped with an electrospray ionization source operating in positive mode and negative mode. The optimal conditions were set as follows: source temperature at 400 ℃, sheath gas flow rate at 40 arb, Aux gas flow rate at 10 arb, ion-spray voltage floating at -2800V in negative mode and 3500V in positive mode, respectively, Normalized collision energy, 20-40-60V rolling for MS/MS. Data acquisition was performed with the Data Dependent Acquisition mode. The detection was carried out over a mass range of 70-1050 m/z.

The pretreatment of LC/MS raw data was performed by Progenesis QI (Waters Corporation，Milford, USA) software, and a three-dimensional data matrix in CSV format was exported. The information in this three-dimensional matrix included: sample information, metabolite name and mass spectral response intensity. Internal standard peaks, as well as any known false positive peaks (including noise, column bleed, and derivatized reagent peaks), were removed from the data matrix, deredundant, and peak-pooled. At the same time, the metabolites were identified by searching the database, and the main databases were the HMDB (http://www.hmdb.ca/), Metlin (https://metlin.scripps.edu/), and the self-compiled Majorbio Database (MJDB) of Majorbio Biotechnology Co., Ltd (Shanghai, China). The data matrix obtained by searching the database was uploaded to the Majorbio cloud platform (https://cloud.majorbio.com) for data analysis. Firstly, the data matrix was pre-processed, as follows: At least 80% of the metabolic features detected in any set of samples were retained. After filtering, the minimum value in the data matrix was selected to fill the missing value, and each metabolic signature was normalized to the sum. To reduce the errors caused by sample preparation and instrument instability, the response intensities of the sample mass spectrometry peaks were normalized using the sum normalization method, to obtain the normalized data matrix. Meanwhile, the variables of QC samples with relative standard deviation (RSD) > 30% were excluded and log10 logarithmicized to obtain the final data matrix for subsequent analysis. Perform variance analysis on the matrix file after data preprocessing.

The R package “ropls” (Version 1.6.2) was used to perform partial squares discriminant analysis (PLS-DA) and orthogonal partial least squares discriminant analysis (OPLS-DA), and 7-cycle interactive validation evaluating the stability of the model. The metabolites with VIP > 1, *p*-value < 0.05 were determined as significantly different metabolites based on the Variable importance in the projeciton (VIP) obtained by the OPLS-DA model and the *p*-value generated by student’s t test. Differential metabolites among two groups were mapped into their biochemical pathways through metabolic enrichment and pathway analysis based on KEGG database (<http://www>.genome.jp/kegg/). These metabolites could be classified according to the pathways they are involved or the functions they performed. Enrichment analysis was used to analyze a group of metabolites in a function node whether appears or not. The principle was that the annotation analysis of a single metabolite develops into an annotation analysis of a group of metabolites. Python packages “scipy.stats” (https://docs.scipy.org/doc/scipy/) was used to perform enrichment analysis to obtain the most relevant biological pathways for experimental treatments. Heatmap were drawn with the "pheatmap" package of R software.

**Gut microbiota**

Three groups, each consisting of 6 mice, were used. DNA was obtained from cecum contents with the Fast DNA SPIN extraction kit (MP Biomedicals, Santa Ana, CA, USA). Primers 338F (5′-ACTCCTACGGGAGG CAGCAG-3′) and 806R (5′-GGACTACHVGGGTWTCTAAT-3′) were used to amplify the V3-V4 region of the 16S rRNA gene by PCR. On the Illumina MiSeq platform (Illumina, San Diego, CA, U.S.A.) at Majorbio (Shanghai, China), high-throughput sequencing was run. Raw FASTQ files were de-multiplexed using an in-house perl script, and then quality-filtered by fastp version 0.19.6 [5] and merged by FLASH version 1.2.7 [6] with the following criteria: (i) the reads were truncated at any site receiving an average quality score of <20 over a 50 bp sliding window, and the truncated reads shorter than 50 bp were discarded, reads containing ambiguous characters were also discarded; (ii) only overlapping sequences longer than 10 bp were assembled according to their overlapped sequence. The maximum mismatch ratio of overlap region is 0.2. Reads that could not be assembled were discarded; (iii) Samples were distinguished according to the barcode and primers, and the sequence direction was adjusted, exact barcode matching, 2 nucleotide mismatch in primer matching. Then the optimized sequences were clustered into operational taxonomic units (OTUs) using UPARSE 7.1 [7] with 97% sequence similarity level. The most abundant sequence for each OTU was selected as a representative sequence. To minimize the effects of sequencing depth on alpha and beta diversity measures, the number of 16S rRNA gene sequences from each sample was rarefied to 20,000, which still yielded an average Good’s coverage of 99.09%, respectively. The taxonomy of each OTU representative sequence was analyzed by RDP Classifier version 2.2 [8] against the 16S rRNA gene database (eg. Silva v138) using confidence threshold of 0.7. PICRUSt2 is a software package designed for predicting functional profiles from 16S amplicon sequencing data [9]. It aligns representative sequences (16S rRNA) against reference sequences in the Integrated Microbial Genomes database to construct a phylogenetic tree. This process identifies the nearest neighbors with sequenced genomes. PICRUSt2 then predicts the functional composition of the microbial community based on the gene type and abundance information of known species. Here, we utilized PICRUSt2 in conjunction with the KEGG database (<http://www>.genome.jp/kegg/) to infer the abundance of functional enzymes. Raw reads were saved in the Sequence Read Archive (SRA) database as NCBI accession PRJNA896555.

***C. elegans* and experimental design**

Caenorhabditis Genetics Center (CGC) gave us three strains: N2 (wild-type), GR1366 (*mgls42 [tph-1::GFP+pRF4(rol-6(su1006))]*) and EU31 (*skn-1(zu135)IV*). At 22 °C, worms were kept on plates of nematode growth media (NGM) with *E. coli* OP50. Eggs were obtained by hypochlorite lysis of nematodes. Eggs, excluding the Control group, were subjected to copper (1.0 mg/L) in the presence or absence of *W. coagulans* XY2 (2 × 10^7^ CFU/mL) [10]. 3 days later, synchronized nematodes were used to perform the following experiments, with each group tested for at least 30 nematodes per experiment.

**Movement assay**

Head swing refers to the frequency of the head moving from side to side and returning in a span of 60 seconds. Body bend is the number of times the middle of the body changes direction of flexion in 1 minute. 30 nematodes were examined per treatment.

**Associative memory assay**

Two points, P and Q, were 4 cm apart. At Q, 1 cm^3^ NGM medium containing 100 mM NaCl was added at Q. The 9 cm NGM petri dish was refrigerated at 4 ℃ overnight to create a NaCl gradient. The medium was taken out 15 minutes prior to positioning hungry nematodes at point S, 3 cm away from P and Q. After 1 h, nematode counts were taken in circles with a 3 cm diameter centered on points P and Q. The difference in nematode counts between the two circles was divided by the total count to determine the chemotaxis index (CI) for NaCl. At least 100 nematodes were present in each group, and six replicates were conducted.

**Chemotaxis assay**

Add 5ul of an odorous substance and solvent in locations P and Q equidistant from the center of the 9 cm NGM petri dish, then add the nematode at the center point S. After 1 h, nematode counts were taken in circles with a 3 cm diameter centered on points P and Q. The difference in nematode counts between the two circles was divided by the total count to determine the CI, where a positive value indicates that the odorant is attractive to the nematode and a negative value indicates aversion. At least 100 nematodes were present in each group, and six replicates were conducted.

**Evaluation of intestinal barrier integrity**

Brilliant blue is used as an indicator for assessing the integrity of the nematode intestinal barrier because it can only penetrate the damaged intestinal epithelium. The nematodes were immersed in *E. coli* OP50 containing 5.0% brilliant blue at 20 ℃ for 3 h, and then were washed with M9 buffer for clarification. After anesthesia, the nematodes were photographed with a microscope to observe the distribution of brilliant blue in the nematode intestine. The proportion of the blue area was calculated using the ImageJ software. 30 nematodes were examined per treatment.

**Evaluation of neuronal development**

We assessed serotonergic neurons in transgenic GR1366 strains. After a 3-day exposure, the nematodes were anesthetized and the green fluorescence was visualized under the FITC channel in a Nikon TiE fluorescent microscope. The exposure time is 1 s. We classified damage as either normal (intact neurons) or abnormal (missing or disconnected neurons). Experiments were conducted in triplicate, and at least 30 nematodes were observed each time.

**Biochemical analysis**

Take 100 mg of mouse brain or colon tissue, add pre-cooled physiological saline at a ratio of 1:9 (w/v), and homogenize with a tissue homogenizer in an ice bath (30 Hz, 5 min). The homogenate was centrifuged (12,000 × g, 10 min, 4°C), and the supernatant was collected for subsequent assays. The activities of glutathione peroxidase (GSH-Px), glutathione-reductase (GR), catalase (CAT), superoxide dismutase (SOD), and levels of glutathione (GSH) in the mouse brain and colon were determined using commercial kits according to the manufacturer’s instructions (Jiancheng Institute of Bioengineering, Nanjing, China). Serotonin (5-HT) levels were measured using 5-HT ELISA Kit (E-EL-0033, Elabscience, USA). Protein content normalization was achieved using BCA Kit (Beyotime, Shanghai, China). Brain tissues were subjected to wet digestion using concentrated HNO_3_ followed by analysis for copper using atomic absorption spectroscopy. 8 mice were examined per treatment.

**qRT-PCR**

Total RNA was extracted from mouse brain and colon samples using RNAiso Plus (TaKaRa, Japan), followed by cDNA synthesis using TransScript All-in-One First-Strand cDNA Synthesis SuperMix (Transgen Biotech, Beijing, China). The primer sequences utilized in this study can be found in Table S1. Real-time PCR was performed using Hieff^®^ qPCR SYBR Green Master Mix (YEASEN, Shanghai, China) with PCR conditions of 95 °C for 5 min, followed by forty cycles at 95 °C for 10 s and 60 °C for 30 s. β-actin was employed as a reference gene. 4 mice were examined per treatment.

**Statistical analysis**

Mean values with standard error of the mean were displayed, and visual representations were created with GraphPad Prism software (version 8.0.1, GraphPad Software, San Diego, CA, USA). Statistical comparisons were performed as follows: unpaired two-tailed Student's t-test for comparisons between two groups, with significant differences (*p* < 0.05) denoted by *; one-way ANOVA followed by Tukey test for multi-group comparisons, with significant differences (*p* < 0.05) indicated by different letters (a, b, c). The fluorescence intensity was analyzed using ImageJ software (version 1.52, National Institutes of Health, Bethesda, MD, USA).

**Figure S1 Copper caused metabolic disorders in the mouse brain.** (A) PLS-DA score plot in positive ion mode. (B) KEGG Compound classification. The vertical axis shows KEGG compound classification, and the horizontal axis shows the number of compounds in each category, with bar color indicating primary classification. (C) Volcano plot of brain differential metabolites between CuSO_4_ group and Control group, as well as between XY2 group and CuSO_4_ group. (D) Clustering heatmap and VIP scores of the top30 differential metabolites between Control and CuSO_4_ groups. Asterisk represented a significant difference between CuSO_4_ group and XY2 group (**p* < 0.05, ** *p* < 0.01, *** *p* < 0.001, unpaired two-tailed Student's t-tests, *n* = 6).

**Figure S2 *W. coagulans* XY2 modulated copper-induced metabolic disorders in the mouse brain.** (A) Pie chart of HMDB superclass. (B) KEGG pathways. The vertical axis represents KEGG pathways, the horizontal axis shows the number of compounds in each category, and the color of the bar indicates the primary classification of the pathway. (C) KEGG Topology Analysis Bubble Diagram. The larger the bubble, the more important the pathway. *n* = 6.

**Figure S3 Tph metabolism-related parameters.** (A) Volcano plot of serum differential metabolites between CuSO_4_ group and Control group, as well as between XY2 group and CuSO_4_ group. *n* = 6. (B) Abundance of *Bacteroides*, *Closteidium_sensu _stricto_1*, *Bifidobacterium* and *Akkermansia.* Different letters represented significant difference among experimental groups, while the same letters represent no significant differences (*p* < 0.05, one-way ANOVA followed by Tukey test, *n* = 5). (C) Abundance of Arylformamidase (EC 3.5.1.9), Kynureninase (EC 3.7.1.3), Tryptophan synthase (EC 4.2.1.20), Tryptophanase (EC 4.1.99.1), and Cinnamoyl-CoA: phenyllactate CoA-transferase (EC 2.8.3.17). Different letters represented significant difference among experimental groups, while the same letters represent no significant differences (*p* < 0.05, one-way ANOVA followed by Tukey test, *n* = 5).

**Figure S4 *W. coagulans* XY2 attenuated copper-induced oxidative stress in the brain and colon of mice.** (A) Enzyme activities of GSH-Px, GR, CAT, and the level of GSH in the mouse brain. Different letters represented significant difference among experimental groups, while the same letters and ns represent no significant differences (*p* < 0.05, one-way ANOVA followed by Tukey test, *n* = 8). (B) Enzyme activities of GSH-Px, GR, CAT, and the level of GSH in the mouse colon. Different letters represented significant difference among experimental groups, while the same letters and ns represent no significant differences (*p* < 0.05, one-way ANOVA followed by Tukey test, *n* = 8). (C) Representative images of O_2_^-^ levels in colon tissue and the relative fluorescence intensity of O_2_^-^. Scale bars = 200 μm. Different letters represented significant difference among experimental groups, while the same letters represent no significant differences (*p* < 0.05, one-way ANOVA followed by Tukey test, *n* = 8).

**Figure S5 mRNA expression levels of intestinal barrier-related genes.** Different letters represented significant differences among experimental groups, while the same letters and ns represent no significant differences (*p* < 0.05, one-way ANOVA followed by Tukey test, *n* = 4).

**Figure S6 *W. coagulans* XY2 inhibited copper-induced neurodegenerative movement disorders in N2 nematodes.** (A) Head swing frequency. Different letters represented significant differences among experimental groups, while the same letters represent no significant differences (*p* < 0.05, one-way ANOVA followed by Tukey test, *n* = 30). (B) Body bends frequency. Different letters represented significant differences among experimental groups, while the same letters represent no significant differences (*p* < 0.05, one-way ANOVA followed by Tukey test, *n* = 30).

**REFERENCES**

1. Huang, Xuedi, Fang Ai, Chen Ji, Pengcheng Tu, Yufang Gao, Yalan Wu, Fujie Yan, Ting Yu. 2021. “A rapid screening method of candidate probiotics for inflammatory bowel diseases and the anti-inflammatory effect of the selected strain Bacillus smithii XY1.” *Frontiers in Microbiology* 12: 760385. <https://doi.org/10.3389/fmicb.2021.760385>

2. Saenkham-Huntsinger, Panatda, Amanda N Hyre, Braden S Hanson, George L Donati, L Garry Adams, Chanelle Ryan, Alejandra Londoño, et al. 2021. “Copper resistance promotes fitness of methicillin-resistant Staphylococcus aureus during urinary tract infection.” *MBio* 12: 10-1128. <https://doi.org/10.1128/mbio.02038-21>

3. Wilhelm, Jiří, Richard Vytášek, Ivana Ošťádalová, Luděk Vajner. 2009. “Evaluation of different methods detecting intracellular generation of free radicals.” *Molecular and Cellular Biochemistry* 328: 167-176. <https://doi.org/10.1007/s11010-009-0086-5>

4. Qi, Meng-han, Hai-yan Zhang, Yun-yi Hou, Ivan Steve Nguepi Tsopmejio, Wei Liu, Wen-guang Chang, Chen Chen, Zi Wang, Wei Li. 2025. “Ginseng-derived GABAFG ameliorates type 2 diabetes mellitus by modulating autophagy-lysosome pathway and gut microbiota.” *Journal of Advanced Research* <https://doi.org/10.1016/j.jare.2025.01.003>

5. Chen, Shifu, Yanqing Zhou, Yaru Chen, Jia Gu. 2018. “fastp: an ultra-fast all-in-one FASTQ preprocessor.” *Bioinformatics* 34: i884-i890. <https://doi.org/10.1093/bioinformatics/bty560>

6. Magoč, Tanja, Steven L Salzberg. 2011. “FLASH: fast length adjustment of short reads to improve genome assemblies.” *Bioinformatics* 27: 2957-2963. <https://doi.org/10.1093/bioinformatics/btr507>

7. Edgar, Robert C. 2013. “UPARSE: highly accurate OTU sequences from microbial amplicon reads.” *Nature methods* 10: 996-998. <https://doi.org/10.1038/nmeth.2604>

8. Wang, Qiong, George M Garrity, James M Tiedje, James R Cole. 2007. “Naive Bayesian classifier for rapid assignment of rRNA sequences into the new bacterial taxonomy.” *Applied and Environmental Microbiology* 73: 5261-5267. <https://doi.org/10.1128/AEM.00062-07>

9. Douglas, Gavin M, Vincent J Maffei, Jesse R Zaneveld, Svetlana N Yurgel, James R Brown, Christopher M Taylor, Curtis Huttenhower, Morgan GI Langille. 2020. “PICRUSt2 for prediction of metagenome functions.” *Nature Biotechnology* 38: 685-688. <https://doi.org/10.1038/s41587-020-0548-6>

10. Zhang, Ying, Chao Zhao, Hu Zhang, Ran Liu, Shizhi Wang, Yuepu Pu, Lihong Yin. 2021. “Integrating transcriptomics and behavior tests reveals how the C. elegans responds to copper induced aging.” *Ecotoxicology and Environmental Safety* 222: 112494. <https://doi.org/10.1016/j.ecoenv.2021.112494>
